# Denosumab: A potential new treatment option for recurrent Aneurysmal Bone Cyst of the spine

**DOI:** 10.1051/sicotj/2019007

**Published:** 2019-04-01

**Authors:** Arvind G. Kulkarni, Ankit Patel

**Affiliations:** 1 Mumbai Spine Scoliosis & Disc Replacement Centre, Department of Orthopedics Surgery, Bombay Hospital and Medical Research Center 400002 Mumbai India

**Keywords:** Donesumab, Spine, Aneurysmal bone cyst, Thoracolumbar spine, Aggressive

## Abstract

ABCs are expansile osteolytic lesions typically containing blood-filled spaces separated by fibrous septae. Standard treatment includes surgical resection or curettage and packing; however, for some spinal lesions, the standard approach is not optimal. One therapeutic strategy is to treat spinal ABC with an agent that targets a pathway that is dysregulated in a disease with similar pathophysiology. Denosumab, a human monoclonal antibody to RANKL is effective in the treatment of GCT's. Spinal ABCs are a therapeutic challenge and local recurrence is a concern. We report a case of aggressive recurrent ABC of dorsal spine in a 14-year old female with progressive neurologic deficit who underwent surgical excision and decompression with a recurrence in a short period for which a decompression and fixation was done. She had a recurrence after an asymptomatic period of 6 months and neurologic worsening. Having ruled out use of embolization and radiotherapy, a remission was achieved by treatment with Denosumab using the regimen for GCTs for a duration of 6 months. Follow-up MRI and CT scans at 24 months following inception of Denosumab depicted complete resolution and no recurrence. We conclude that Denosumab can result in symptomatic and radiological improvement in the recurrent locally aggressive ABC and may be useful in selected cases. Long-term results are mandatory to confirm the efficacy of Denosumab and to evaluate local recurrence after stopping Denosumab.

## Introduction

ABCs are expansile lesions accounting for approximately 15% of all primary spinal tumours [1–3]. Multiple treatment modalities like intra-lesional excision, Selective Arterial Embolisation, injection with sclerosing-agents and radiation have been attempted with variable improvement and recurrence rates. However, these approaches may be associated with severe morbidities, where lesion is in the thoracic spine [4,5].

Giant-cells that occur in ABCs are positive for markers of true osteoclasts [6,7]. The osteoclast secretes receptor activator of nuclear factor k-B ligand (RANKL) [8–12]. Here, we report a case of recurrent ABC of the thoracic-spine that achieved resolution after treatment with Denosumab, a human monoclonal-antibody to RANKL that is effective in the treatment of GCTs [9,10,12] and ABCs [6,13–16].

## Case report

**Figure 1.**
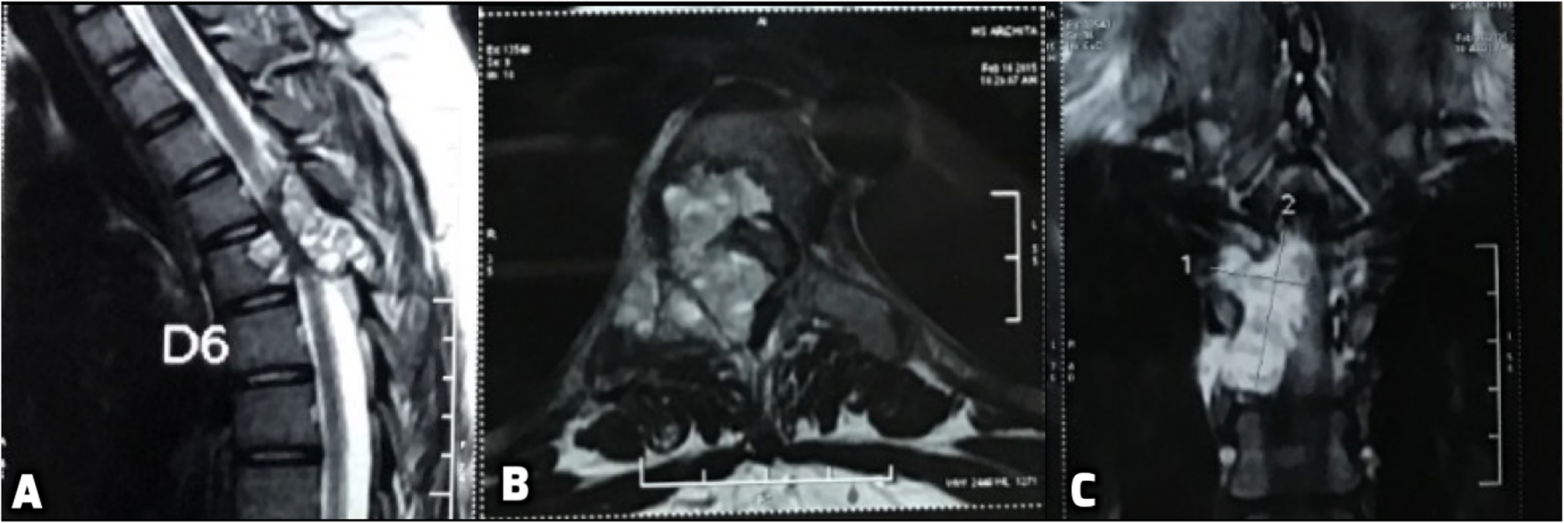
First Pre-operative MRI (A) revealing an intensely enhancing cystic cum haemorrhagic septate expansile bony lesion (5.7 × 3.1 cm) involving the posterior part of D5 vertebral body, right lamina, right pedicle and transverse process with epidural extension (D4–D5) causing marked spinal cord compression and underlying cord oedema. (B) Axial T2 MRI scan depicts the encroachment of the right half of body and posterior elements with compression of the spinal cord. (C) Coronal image through the spinal canal showing intense enhancement on T2 image with fluid levels.

**Figure 2.**
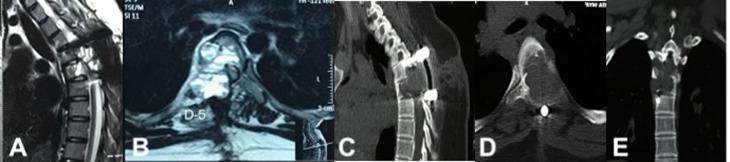
Post-operative day 2 MRI showing surgical mass compressing dorso-laterally from right (A, B), CT scan depicting the extent of bony destruction (C–E) with pedicle screw fixation and excision of ABC.

**Figure 3.**
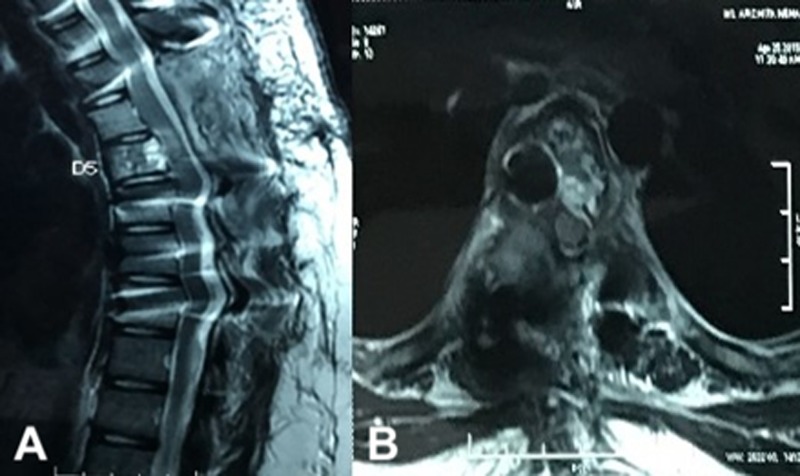
Post second intra-lesional resection MRI showing removal of epidural compressive mass. Dorsal spinal cord decompressed ventro-dorsally on sagittal (A) and axial views (B) No evidence of enhancing fluid filled lesion on T2WI of MRI.

**Figure 4.**
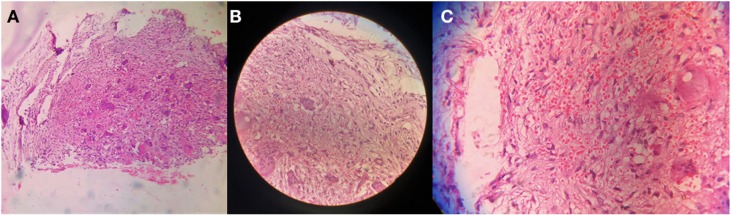
Hematoxylin and eosin-stained slide, scanner view showing Cystic spaces surrounded by fibrous tissue proliferation along with multiple osteoclastic giant cells at 4× magnification (A). 10× magnification (B) and 40× magnification (C) showing cystic spaces filled with few rbcs lined by bland fibroblast and dense fabricollagenous tissue in the surrounding with proliferating capillaries and numerous osteoclastic giant cells.

**Figure 5.**
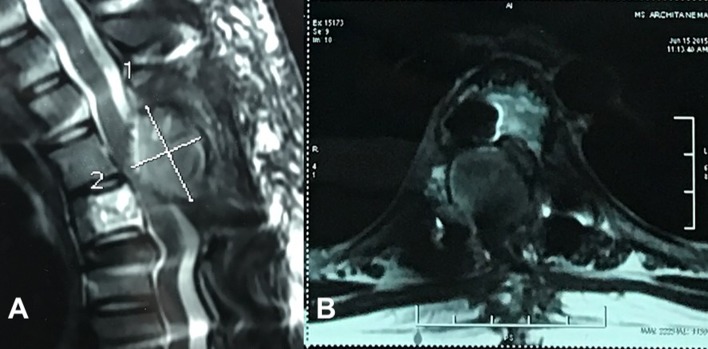
MRI after second recurrence showing Epidural compressive mass (cross-hairs) from the right side with underlying Myelomalacia of the spinal cord with ventral displacement on sagittal (A) and axial scans (B).

A 14-year-old female presented with unsteady gait and back-pain. She had undergone two previous spinal surgeries in the recent past at another hospital. The previous medical-records showed that the girl developed insidious-onset, progressive spastic sensory-motor paraparesis. MRI of the dorsal-spine (Figure 1) suggested an intensely enhancing cystic-haemorrhagic septate expansile lesion (5.7 × 3.1 cm) involving D5 vertebra with epidural extension (D4–D5) causing marked spinal-cord compression and oedema. The lesion was hypo-hyperintense on T2WI, with intense heterogeneous enhancement. T1 hyperintense signals suggested haemorrhagic component. SAE was ruled out by the interventional neuro-radiologist due to potential risk of vascular insult to the spinal-cord. An intra-lesional excision was done and D4–D6 unilateral pedicle screw-rod fixation was performed. After 2 days of initial neurological improvement, the patient developed neurological worsening, which was attributed to a compressive surgical mass. Following re-exploration and removal (Figure 2), the patient had recovery for a period of 2 months, but again worsened neurologically. MRI revealed an increase in the epidural mass with spinal cord-compression. The patient presented to the authors at this stage. A revision decompression was performed and circumferential reconstruction was done with a mesh-cage and bilateral pedicle-screw-rod fixation from D1 to D8. She made a significant neurologic recovery (Figure 3). Histopathology revealed an ABC (Figure 4). After 3 months, the patient had recurrent symptoms again in the form of back-pain and unsteadiness of gait that was explained by an epidural lesion (3.4 × 2.5 cm) compressing the spinal cord with myelomalactic changes (Figure 5). The situation had a significant negative psychological impact on the young girl. Alternative treatment options were sought and finally treatment with Denosumab was offered to the patient after thorough counselling regarding the expected benefits, experimental nature and possible adverse-effects.

**Figure 6.**
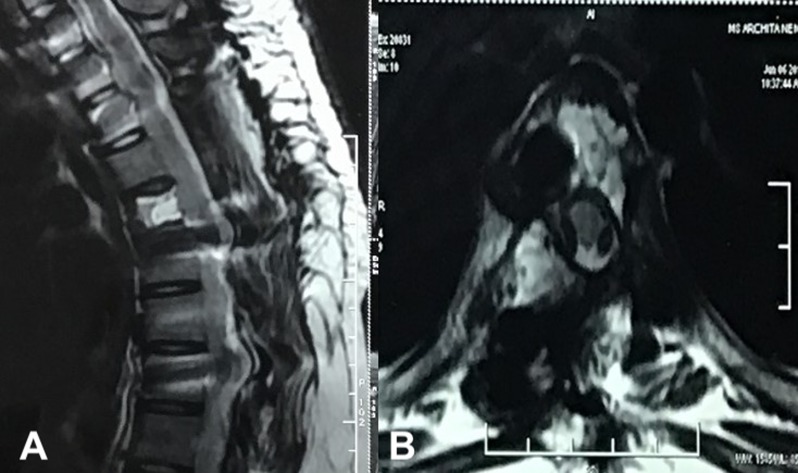
A 6-month follow-up MRI post completion of Denosumab therapy. (A) shows a sagittal T2WI MRI image with resolution of previous dorsal compressive mass and (B) axial image of the decompressed spinal cord.

**Figure 7.**
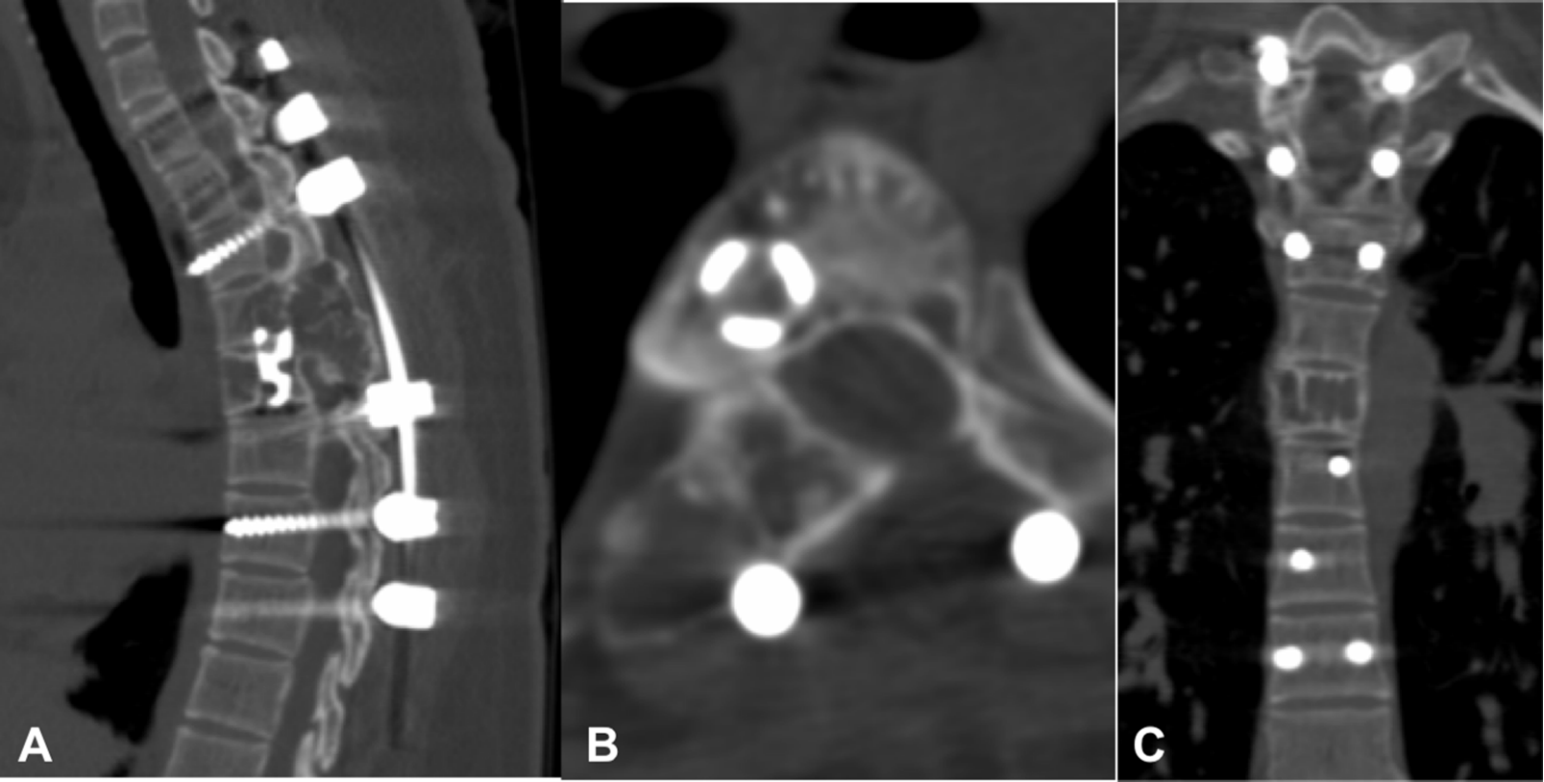
2-year follow-up CT scan. Ossification of the lesion with remodelling of the spinal canal and incorporation of Harm’s cage seen on Sagittal (A), axial (B) and Coronal (C) images.

Treatment was begun using the regimen described for treating GCT [9–11] (120 mg sc D1, 8, 15, 28 and then monthly). She improved within a month’s time. MRI at 2 months of therapy documented significant resolution of epidural compressive mass and MRI at 6 months depicted complete resolution (Figure 6). Denosumab treatment was discontinued at 6 months and scans at 1 and 2 years showed no recurrence. CT at 2 years showed ossification of the lesion with remodelling of the spinal canal and incorporation of the mesh-cage (Figure 7).

## Discussion

ABCs are generally rare tumorous lesions of the spine, but can lead to devastating symptoms as they are capable of destructive growth. Common treatment options for ABCs include surgical resection, curettage and filling, selective arterial embolisation, total arterial embolisation or radiotherapy. For spinal ABCs, however, some of these options entail considerable risks. Sclerosant therapy was not considered in our patient as the cortices were eroded following previous surgical excisions, and hence there was a risk of seepage of sclerosant into neuro-vascular elements leading to catastrophic complications [13].

Denosumab, a human monoclonal-antibody, binds to a cytokine receptor activator of nuclear factor-kappa Bligand (RANKL) and prevents the action of agonists acting through RANKL-receptors. This prevents the subsequent activation and proliferation of the osteoclasts [14]. Seven patients have been successfully treated with Denosumab in addition to our patient [6,13–15,17] (Table 1). Clinical trials have shown that Denosumab is effective in reducing tumour mass in case of refractory, recurrent or inoperable GCT [9,10]. Denosumab also inhibits normal bone remodelling and an important toxicity of Denosumab is osteonecrosis of the jaw [18–20]. Again, osteopetrosis or osteosclerosis after use of Denosumab in young patients is possible [21–23].

**Table 1.** Published and current cases of Denosumab treatment of ABC.

	Case 1	Case 2	Case 3	Case 4	Case 5	Case 6	Case 7	Current case
Age/sex	8-year male	11-year male	5-year male	27-year male	26-year female	42-year male	16-year male	14-year female
Location	C5	C5	Sacrum	Sacrum	C7–T1	L5–S1	L5	D5
Diagnosis	Typical ABC	Typical ABC	Typical ABC	Typical ABC	Typical ABC	Aggressive ABC	Typical ABC	Typical ABC
Initial treatment	Surgery	Surgery	None	None	C4–T4 fixation, cervical laminectomy	Seven consecutive SAE	Five consecutive monthly SAEs	Surgery twice
Outcome of initial treatment	Recurrence at 1 month	Recurrence at 8 months	NA	NA	NA (adjuvant therapy)	Recurrence at 6 months, worsening radiculopathy	Pathologic vascularity after embolisation, right sciaticalgia	Recurrence at 2 months
Denosumab treatment	70 mg/m^2^ sc monthly	70 mg/m^2^ sc D1, 8, 15, 21, 28 and then monthly	1.2 mg/kg/dose sc D1, 8, 15, 21, 28 then monthly	Denosumab 120 mg sc D1, 8, 15, 28 and then monthly for 1 yr., followed by observation	120 mg sc monthly for 16 months	120 mg once a week for 4 weeks consecutively, then once every 40 days, 13 Denosumab administrations	120 mg once a week for 4 weeks consecutively, then once every 40 days, 11 Denosumab administrations	Denosumab 120 mg sc D1, 8, 15, 28 and then monthly for 6 months, followed by observation
Response to Denosumab	Recovery from neurological symptoms, and no progression of ABC, with evidence of bone formation on MRI at 2 months	Recovery from neurological symptoms, and no progression of ABC, with evidence of bone formation on MRI at 4 months	Resolution of pain and new bone formation with healing of pathologic fracture on MRI	Resolution of pain and new bone formation on CT scan, decrease in giant cells, and decrease in cellularity of fibrous stroma	Persistence of C8–T1 deficit, peripheral bone reconstruction, decrease in tumour size and bone fusion at 19.5 months follow-up	Complete ossification at 35 months CT scan. Pain-free	gradual mass reduction and progressive ossification since the 3rd month of administration. 33 months follow-up	Resolution of pain and new bone formation on MRI scan, complete recovery of neurologic worsening. No recurrence at 1.5 year follow-up
Follow-up	2 months	4 months	12 months	12 months	19.5 months	35 months	33 months	24 months
References	Lange et al. [6]	Lange et al. [6]	Pelle et al. [17]	Skubitz et al. [13]	Arnaud Dubory et al. [15]	Ghermandi et al. [14]	Ghermandi et al. [14]	

*Note*: ABC indicates aneurysmal bone cyst; SAE, Selective Arterial Embolisation; NA, not applicable; CT, computed tomographic; MRI, magnetic resonance image; sc, sub-cutaneous.

Denosumab has beneficial effects as in case of recurrent ABC described in this report. Surgical endeavours had failed twice and other proven modalities were ruled out. Previous failed surgeries had left the surgeons as well as the patient and relatives in despair with low hopes of further surgeries and in the outlook for newer horizons. We were unable to find a similar report of an aggressive refractory ABC of the dorsal spine in which deployment of Denosumab was successful.

## Conclusion

We conclude that the usage of Denosumab, although based on anecdotal experiences, may be an excellent option in cases not amenable to surgical interventions and frequent recurrences. Long-term results should confirm these preliminary outcomes to standardise the treatment-guidelines.
